# Cloning and Functional Studies of a Splice Variant of CYP26B1 Expressed in Vascular Cells

**DOI:** 10.1371/journal.pone.0036839

**Published:** 2012-05-29

**Authors:** Ali Ateia Elmabsout, Ashok Kumawat, Patricia Saenz-Méndez, Olesya Krivospitskaya, Helena Sävenstrand, Peder S. Olofsson, Leif A. Eriksson, Åke Strid, Guro Valen, Hans Törmä, Allan Sirsjö

**Affiliations:** 1 Department of Clinical Medicine, School of Health Sciences, Örebro University, Örebro, Sweden; 2 Computational Chemistry and Biology Group, Facultad de Química, UdelaR, Montevideo, Uruguay; 3 Department of Science and Technology, Örebro Life Science Center, Örebro University, Örebro, Sweden; 4 Department of Medicine, Karolinska Institutet, Center for Molecular Medicine, Stockholm, Sweden; 5 Laboratory of Biomedical Science, The Feinstein Institute for Medical Research, North Shore-LIJ Health System, Manhasset, New York, United States of America; 6 Department of Chemistry and Molecular Biology, University of Gothenburg, Göteborg, Sweden; 7 Department of Physiology, Institute of Basic Medical Science and Center for Heart Failure Research, University of Oslo, Oslo, Norway; 8 Department of Medical Sciences, Dermatology and Venereology, Uppsala University, Uppsala, Sweden; Cardiovascular Research Institute Maastricht, Maastricht University, The Netherlands

## Abstract

**Background:**

All-trans retinoic acid (atRA) plays an essential role in the regulation of gene expression, cell growth and differentiation and is also important for normal cardiovascular development but may in turn be involved in cardiovascular diseases, i.e. atherosclerosis and restenosis. The cellular atRA levels are under strict control involving several cytochromes P450 isoforms (CYPs). CYP26 may be the most important regulator of atRA catabolism in vascular cells. The present study describes the molecular cloning, characterization and function of atRA-induced expression of a spliced variant of the *CYP26B1* gene.

**Methodology/Principal Findings:**

The coding region of the spliced *CYP26B1* lacking exon 2 was amplified from cDNA synthesized from atRA-treated human aortic smooth muscle cells and sequenced. Both the spliced variant and full length CYP26B1 was found to be expressed in cultured human endothelial and smooth muscle cells, and in normal and atherosclerotic vessel. atRA induced both variants of CYP26B1 in cultured vascular cells. Furthermore, the levels of spliced mRNA transcript were 4.5 times higher in the atherosclerotic lesion compared to normal arteries and the expression in the lesions was increased 20-fold upon atRA treatment. The spliced CYP26B1 still has the capability to degrade atRA, but at an initial rate one-third that of the corresponding full length enzyme. Transfection of COS-1 and THP-1 cells with the CYP26B1 spliced variant indicated either an increase or a decrease in the catabolism of atRA, probably depending on the expression of other atRA catabolizing enzymes in the cells.

**Conclusions/Significance:**

Vascular cells express the spliced variant of *CYP26B1* lacking exon 2 and it is also increased in atherosclerotic lesions. The spliced variant displays a slower and reduced degradation of atRA as compared to the full-length enzyme. Further studies are needed, however, to clarify the substrate specificity and role of the CYP26B1 splice variant in health and disease.

## Introduction

Retinoids are important for normal cardiovascular development [1] but may also be involved in cardiovascular diseases, i.e. atherosclerosis and restenosis [2]–[4]. Especially the ability of retinoids to reduce inflammation and proliferation could be of importance for the development of cardiovascular diseases.

Biologically active retinoid metabolites are synthesized in target cells from all-*trans* retinol (atROH) taken up from the circulation [5]. There are several microsomal CYP enzymes which are suggested to be involved in retinoid metabolism, e.g. CYP1A1, CYP4A11, CYP3A4/5/7 and CYP2C8/9 [6], [7]. Most important, however, seems to be the CYP26, which is responsible for catabolism of retinoic acid. It regulates intracellular levels of atRA and degrades it into inactive derivatives or polar metabolites such as 4-OH-RA, 4-oxo-RA, 5, 8-epoxy-RA and 18-OH-RA [8]–[11]. CYP26 recognizes atRA as its substrate and its expression and/or its activity can be induced by RA both *in vitro* and *in vivo* [10], [12]–[14]. Three isoforms of CYP26 exist in humans: CYP26A1, CYP26B1 and CYP26C1 [8], [10], [15], [16]. CYP26B1 converts all-*trans*-RA (atRA) to different polar metabolites with a higher efficiency than CYP26A1 [8]. Studies on substrate specificity show that CYP26A1 and B1 are specific for atRA, whereas CYP26C1 efficiently metabolizes both atRA and its isomer 9-*cis*-RA [8], [15]. During treatment of hematopoietic leukemia with RA, the phenomenon of RA-resistance has often been reported [17]. This is probably due to retinoid-induced CYP26 expression resulting in accelerated catabolism of atRA [18]–[20]. Inhibition of CYP26 activity by RA metabolism blocking agents (RAMBAs) increases both plasma and tissue levels of atRA and generates retinoid-like effects [8], [20]. Furthermore, it has been demonstrated that embryos from CYP26-deficient mice exhibit abnormalities that mimic atRA-induced teratogenicity [21]–[23]. Some of the observed abnormalities, however, could also be the result of a deficiency in CYP26-generated metabolites that have active biological roles *in vivo* [21].

*CYP26B1* is highly expressed in intimal smooth muscle cells and up-regulated by lower atRA levels than CYP26A1 [24]. Inhibition of CYP26B1 by R115866 (a synthetic CYP26-inhibitor) increases the levels of atRA in smooth muscle cells [24]. With increased levels of endogenous atRA, a number of retinoid responsive genes are induced, suggesting that CYP26B1 may be a key enzyme in the regulation of retinoid levels in the vessel wall (25). *CYP26B1* was shown to be the major CYP26 expressed and induced by atRA and it is believed to be an important regulator of atRA levels in human vascular cells [24], [25]. We have recently found increased levels of CYP26B1 in atherosclerotic lesions, with the strongest expression found in macrophage-rich, inflammatory areas of the lesions [4]. This localization coincides with the site where it is likely to exert the strongest effect on atRA levels and on the extent of inflammation in the lesion. The CYP26B1 gene consists of six exons and a large second intron of 8.57 kb and covers about 18,000 base pairs (bp) [26]. The gene also has a 3 kb long untranslated 3' region. So far, no spliced variants of *CYP26B1* have been reported.

In this study we describe the cloning and functional studies of an atRA-induced spliced variant of the CYP26B1 gene lacking exon 2. We furthermore investigate the expression of the spliced variant in vascular cells and in atherosclerotic lesions.

## Results and Discussion

### Cloning and Expression of a Splice Variant of CYP26B1

**Figure 1:**
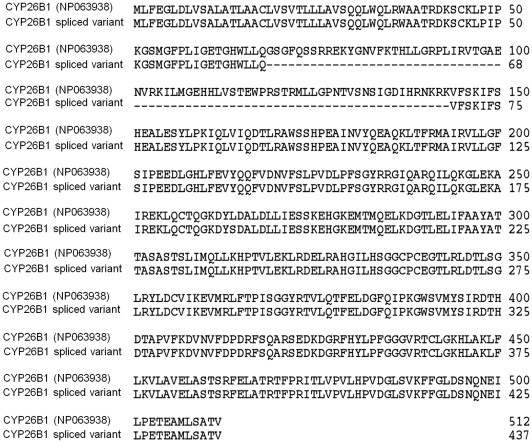
Amino acid sequence of the splice variant *CYP26B1*. Alignment of full length and spliced *CYP26B1* reveals the exclusion of sequence corresponding to exon2 in the latter protein.

Amplification of cDNA from atRA-treated human aortic smooth muscle cells did not only amplify wild type *CYP26B1* but also a shorter spliced variant. Both variants of the *CYP26B1* gene were cloned into the pZErO vector and sequence analysis revealed that exon2 (nt 205–429) was missing in the spliced variant (Fig. 1).

**Figure 2:**
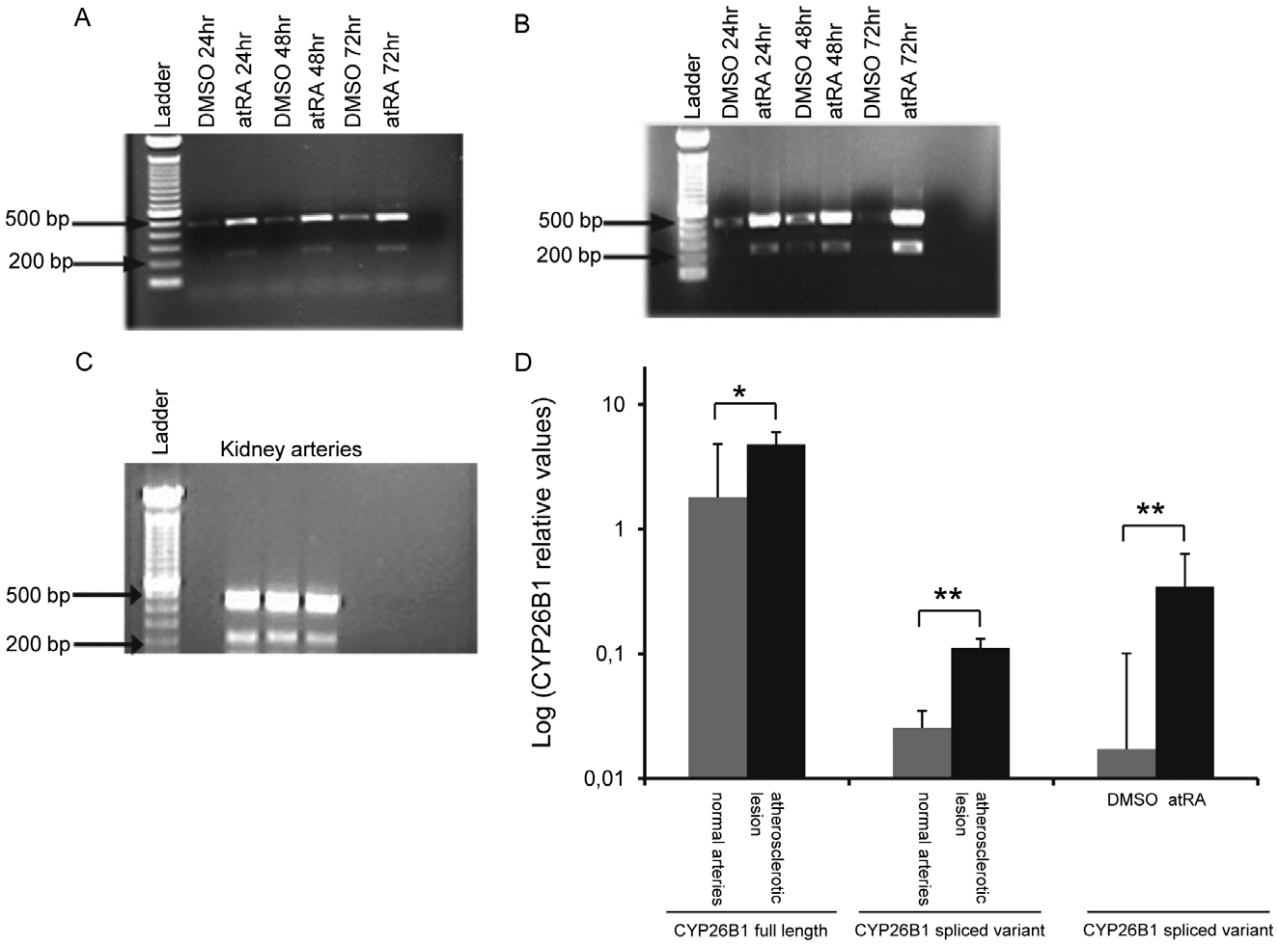
mRNA Levels for the CYP26B1 Spliced Variant in Vascular Cells and in Normal vessels. *CYP26B1* expression in human umbilical vein endothelial cells (HUVECs), aortic smooth muscle cells (AOSMCs) and normal kidney arteries. PCR was performed using primers annealing to exon 1 and exon 3 of *CYP26B1*. In the presence of exon 2 the expected PCR products is 438 bp, in its absence only 213 bp. (A) HUVEC, (B) AOSMC, (C) three independent samples of normal kidney arteries, (D) quantitative RT-PCR analysis of *CYP26B1* transcript levels in atherosclerotic lesion (*n = 6*) and normal kidney arteries (*n = 5*) and transcript levels of CYP26B1 splice variant in atherosclerotic lesion treated with DMSO (n = 4) and atRA (n = 4) (right panel).

**Figure 3:**
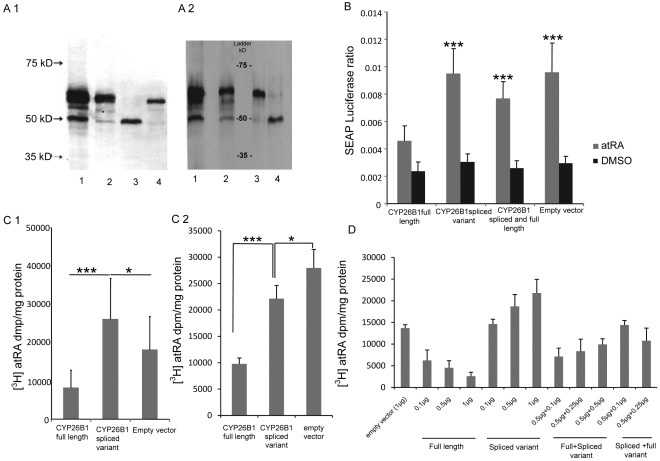
Protein expression of the CYP26B1 splice variant in HUVEC, AOSMCs and COS-1 cells. (A1) Expression of full length and spliced CYP26B1 in atRA-treated human umbilical vein endothelial cells (HUVECs) and (A2) aortic smooth muscle cells (AOSMCs) treated with atRA (lane 1) and DMSO (lane 2) and COS-1 cells transfected with constructs for spliced variant (A1, lane 3, and A2, lane 4) and full length CYP26B1 (A1, lane 4, and A2, lane 3), detected with Western blot analysis. (B) Cell-based reporter pRARE-TA-SEAP was used to measure retinoid levels in the presence of the full length and spliced CYP26B1 in COS-1 cells. SEAP: luciferase ratio in COS-1 cells transfected with either of the two different *CYP26B1* variants, both variants simultaneously, or empty vector after 24 hours treatment with either atRA or DMSO (control). (C1) Cellular concentration of exogenously added [^3^H] atRA in COS-1 and (C2) THP-1 cells transfected with either of the two different *CYP26B1* variants or empty vector. (D4) COS-1 cells transfected with different concentrations of the *CYP26B1* variants separately or in combination. The values are expressed as mean ± S.D., *n = 4*. Statistical significance between *CYP26B1* variants was analysed by Student’s t test and ANOVA, *P<0.05,**P<0.01, ***P<0.001 (spliced variant vs. CYP26B1 full length).

To further investigate whether the *CYP26B1* spliced variant is expressed in vascular cells and in normal kidney arteries, PCR was performed using primers annealing to exon1 and exon 3 of *CYP26B1*. The expected PCR products will be 438 bp in the presence of exon 2, but only 213 bp in its absence. We found that both the full length and the spliced *CYP26B1* were expressed in HUVECs (Fig. 2A), AOSMCs (Fig. 2B) and normal kidney arteries (Fig. 2C). Furthermore, the results show that atRA induced the expression of both *CYP26B1* transcript variants in HUVECs and AOSMCs (Fig. 2A and 2B). To quantify the levels of these transcripts in atherosclerotic and normal vessels, we used real time PCR with specific primers and probes detecting only the full length or only the spliced *CYP26B1*. The transcript levels of full length and the spliced variant of *CYP26B1* was 2.5- and 4.5-fold higher, respectively, in the atherosclerotic lesion compared to normal arteries (Fig 2D). To investigate if atRA could induce the spliced variant in atherosclerotic lesions, we treated atherosclerotic lesions in vitro with 1µM atRA. As seen in Fig. 2D (right panel), atRA induced the spliced variant 20-fold. In previous experiments we found that atRA induced the CYP26B1 full length 7-fold in the atherosclerotic lesions [4]. The corresponding protein expression of full length and spliced CYP26B1 variants, with predicted sizes of 57.5 and 50.2 kDa, respectively, were analyzed with western blot of protein extracts from HUVECs and AOSMCs treated with atRA or DMSO for 48 hours (Fig. 3A1 and 3A2, lane 1 and 2). CYP26B1 splice and full length over-expressed in transfected COS-1 cells was used as positive controls (Fig. 3A, lane 3 and 4 and Fig. 3A2, lane 4 and 3 respectively). It is obvious that expression of both the full length and the spliced CYP26B1 is considerably higher in atRA-treated cells than in controls (Fig. 3A1 and 3A2, lane 1 and 2).

### Metabolic Activity of CYP26B1 Splice Variant

The activity of the spliced CYP26B1 was determined by transfecting COS-1 cells with either full length or spliced CYP26B1 expression constructs, a combination of both, or the corresponding empty vector pcDNA3.2/V5-DEST, together with RARE-SEAP and control luciferase vectors. The results show that the CYP26B1 full length version reduced the RARE-regulated SEAP activity in comparison with cells transfected with the empty vector. Surprisingly, in cells transfected with the spliced variant the SEAP activity was almost the same as in cells transfected with empty vector (Fig. 3B). This implies that the full length CYP26B1 catabolises atRA, resulting in reduced RAR-mediated signaling, which is in agreement with previous studies [25], whereas the spliced CYP26B1 has little or no impact on atRA catabolism. In cells, transfected with a combination of both spliced and full length CYP26B1 (Fig. 3B), the SEAP activity was higher compared with cells transfected with full length CYP26B1 alone.

To further investigate whether the capacity to metabolise RA differs between the full length and spliced variants of CYP26B1, COS-1 cells were transiently transfected with the two expression vectors besides the control vector and incubated with [^3^H]atRA for 1 h (Fig. 3C1). The results show, in agreement with the experiments presented above, that the full length CYP26B1 decreased the cellular levels of atRA compared with cells transfected with empty vector (Fig. 3C1). Interestingly, cells transfected with the spliced variant of CYP26B1 showed an increase in cell-associated [^3^H]atRA compared to cells transfected with full length CYP26B1 and empty vector (Fig. 3C1). This indicates that the spliced variant interferes with endogenous enzymes that catabolise atRA in COS-1 cells, reducing the rate of atRA catabolism. To further explore whether this is a general phenomenon, THP-1 cells that catabolise atRA at a lower rate than COS-1 cells were transfected with the different plasmid constructs. In contrast to COS-1 cells, an increased degradation of atRA by the CYP26B1 splice variant, compared with empty vector alone, was seen (Fig. 3C2). Taken together, this result indicates that the CYP26B1 splice variant could increase or decrease the catabolism of atRA depending on the levels of other atRA catabolizing enzymes in the cells.

To investigate the effect of different levels of *CYP26B1* variants, we transfected COS-1 cells with different concentrations of plasmid construct. As seen in Fig. 3D, transfection with 0.1µg - 1 µg of the *CYP2B1* full length showed a concentration dependent decrease of atRA levels. In contrast, transfection of the cells with *CYP26B1* spliced variant showed a concentration dependent increase of atRA. Transfection of the cells with a mix of *CYP26B1* variants in different concentrations, half amount of *CYP26B1* spliced variant plasmid (ratio1∶2) was needed to partly inhibit the increased degradation seen after transfection with full length *CYP26B1*.

**Figure 4:**
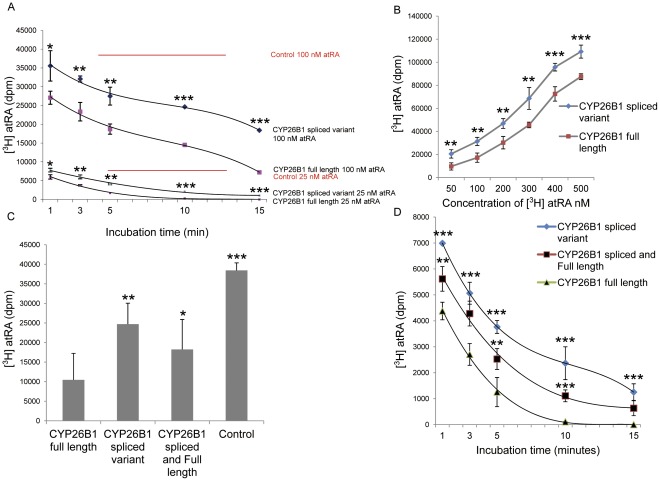
Enzymatic and metabolic assay of CYP26B1. (A) CYP26B1 full length and splice variant were incubated with 25 nM and 100 nM of [^3^H] atRA, respectively, for different time points as indicated. (B) CYP26B1 full length or splice variant was incubated with different concentrations of [^3^H] atRA for 5 minutes and the disappearance of [^3^H] atRA was followed. (C&D) CYP26B1 full length, splice variant, combination of both, and control, incubated with 100 nM of [^3^H] atRA for 10 minutes(C) or incubated with 25nM of [^3^H] atRA for different time points (D) as indicated in figure. The control used in the experiment was either full length CYP26B1 or spliced variant CYP26B1 without initiation by NADPH, the values in the figures are mean ±SD, *n = 3* analyzed by Student’s t-test (Fig4 A &B) and ANOVA (Fig 4C&D),*P<0.05, **P<0.01, ***P<0.001 vs. CYP26B1 full length.

To further investigate the function of the full length and spliced variants of *CYP26B1*, affinity purified proteins were incubated with 25 and 100 nM [^3^H]atRA for different intervals or with different concentrations of atRA for 5 minutes (Fig. 4A-B). The assay shows that the spliced variant of CYP26B1 retained the capability of atRA degradation (Fig. 4A-B), but at a rate that is approximately one-third of the full length enzyme during the first minute, whereafter the rate was almost similar as for the full length enzyme. The large difference in catalytic rate between the full-length and spliced CYP26B1 at the start of the experiment (i.e. at high concentration ≈100 nM of atRA) could either be due to a considerably higher K_m_ for the splice variant or a possible difference in regulation by atRA and its reaction products caused by the deletion.

Moreover, incubating a combination of both variants with 100 nM of [^3^H]atRA for 10 minutes or 25nM of [^3^H]atRA for different intervals, decreased catabolism of atRA compared with the full length enzyme alone (Fig. 4C and D). Our results indicate that the spliced variant possesses a similar affinity for atRA as the full length protein but catabolises it at only one-third of the rate of the full length protein.

### Conclusion

In the present study, we have cloned and characterised a spliced variant of the *CYP26B1* gene which is atRA-inducible in vascular cells. The splice variant shows a slower and reduced degradation of atRA as compared with the full length CYP26B1. One could speculate that the function of the spliced variant may be to reduce available atRA at a lower rate than full length CYP26B1, thereby regulating the rate of atRA degradation. This would prevent a complete depletion of atRA in the cell after induction of *CYP26B1*. Our results also suggest that the effects of the CYP26B1 spliced variant are highly dependent on its relative concentration compared to that of other atRA catabolising enzymes, although its interaction with other co-factors and/or retinoid-binding proteins is yet unknown. Further studies, however, are needed to clarify the substrate specificity and the role of the CYP26B1 splice variant in health and disease.

## Materials and Methods

### Human Biopsies, Tissue Culture, Cell Culture and cDNA Synthesis

Ten patients with atherosclerosis scheduled for carotid endarterectomy and five patients without history of cardiovascular disease scheduled for nephrectomy were included [27]. Six atherosclerotic lesions and five renal arteries were snap-frozen. Four lesions were divided in two and incubated in DMEM/F12 enriched with 30 mg/mL of human albumin (Biovitrum AB, Stockholm, Sweden) for 6 h with 1 µM atRA (Sigma-Aldrich, St. Louis, MO, USA) or vehicle (DMSO). These studies were approved by the regional ethical committee for human studies and subjects were included after informed consent.

Human aortic smooth muscle cells (AOSMCs) and human umbilical vein endothelial cells (HUVECs) were purchased from Cambrex BioScience (Walkersville, Maryland MD, USA) and maintained in recommended growth medium (Cambrex BioScience). HUVECs and AOSMCs at passage 5 to 10 were used throughout the whole study. Confluent AOSMCs and HUVECs cultured in 6-well plates were exposed to 1µM atRA or the vehicle (DMSO) for different time periods.

Total RNA was isolated using the E.Z.N.A., total RNA kit (Omega Bio-Tek, Doraville, GA, USA) according to the manufacturer’s protocol. Finally, 1µg RNA was reverse-transcribed to cDNA using random hexamers and Superscript II reverse transcriptase (Invitrogen, Stockholm, Sweden).

### Cloning and Sequencing of the CYP26B1 Spliced Variant

CYP26B1 was amplified from cDNA synthesized from atRA-treated human AOSMCs. The PCR reaction was carried out with Takara Ex Taq polymerase (Takara Bio Europe, Saint-Germain-en-laye, France) and *CYP26B1* specific primers tagged with recognition sequences for the restriction enzyme *BsrGI*: forward primer (5′- ATG CCA ACT TTG TAC AAA AAA GCA GGC ACC ATG CTC TTT GAG GGC TTG GAT CTG GT-3′), and reverse primer (5′**-** TGC CAA CTT TGT ACA AGA AAG CTG GGT TCT AGA CTG TGG CGC TCA GCAT-3′) (Sigma-Aldrich, St.Louis, MO, USA). The PCR conditions were as follows; 94°C for 2 minutes, followed by 35 cycles at 94°C for 45 seconds, 55°C for 60 seconds, and 72°C for 2 minutes. The PCR products were cloned into the pZErO vector (Invitrogen) and the constructs were sequenced using an ABI PRISM™ 310 Gene Analyzer (Applied Biosystems, Foster City, CA, USA). Both CYP26B1 (full length accession number “NP063938” and splice variant accession number “FJ467289” were sequenced and analysed by using the Lasergene software (DNASTAR Inc, Madison, WI. USA).

*CYP26B1* was sub-cloned into the pcDNA3.2/V5-DEST vector (Invitrogen) and amplified by PCR using the same primers and PCR conditions as described above for the cloning into the pZErO vector. The pZErO plasmid containing the *CYP26B1* spliced gene was used as template. The integrity of the final pcDNA3.2/V-5 DEST-*CYP26B1* spliced construct was confirmed by sequence analysis. *CYP26B1* full length cDNA was recombined with pcDNA3.2/V5-DEST Vector according to the protocol provided by the manufacturer using Gateway^R^ LR Clonase™ II enzyme mix (Invitrogen).

### CYP26B1 Gene Expression

PCR was performed to determine if the *CYP26B1* spliced variant was expressed in AOSMCs, HUVECs and normal vessels. The forward primer (5′-CAC TCG CGA CAA GAG CTG-3′) was located on exon 1 and the reverse primer (5′-GCC TCC TGG TAC ACG TTG AT-3′) on exon 3 (Sigma-Aldrich). The cDNA from normal vessels and atRA- and DMSO-treated AOSMCs and HUVECs were used as the template and AmpliTaq Gold DNA polymerase (Applied Biosystems) was used for amplification. The PCR reaction was initiated at 94°C for 5 min followed by 35 cycles at 95°C for 45s, 57°C for 45s and 72°C for 2 minutes. PCR products were resolved in a 1% agarose gel containing ethidium bromide and the amplicons were monitored under UV-light.

### Quantitative Analysis of CYP26B1mRNA Levels by Real Time PCR

The mRNA levels were determined by using real-time quantitative polymerase chain reaction using TaqMan universal PCR master mix, Assay on demand™ (Applied Biosystems,) for the full-length *CYP26B1*. Primers and probe of the *CYP26B1* spliced variant was designed and obtained from Sigma. The PCR analysis was performed using a 7900HT Fast Real-Time PCR System (Applied Biosystems according to the manufacturer’s instructions). The results were normalized using *Cyclophilin A* (Hs04194521) (Applied Biosystems) as a reference gene.

### Transient Transfection of COS-1 and THP-1 Cells with the CYP26B1 Spliced Variant

COS-1 cells were cultured in Dulbecco’s modified Eagle’s medium (DMEM) 1X (Invitrogen) supplemented with 10% FBS, 2mM L-glutamine and 0.1% penicillin-streptomycin solution in a humidified atmosphere (5% CO_2_, 95% air, 37°C) until they reached 75–85% confluence. Cells were trypsinized and seeded into 6-well plates at 3.2×10^5^ cells/well in DMEM containing 10% FBS and 2mM L-glutamine without antibiotics for 24 hours prior to transfection. COS-1 cells were transfected in duplicates with 2µg plasmid DNA/well of either the *CYP26B1* full length construct, the *CYP26B1* spliced construct or empty pcDNA3.2/V5-DEST vector using 6µl of Lipofectamin 2000 (Invitrogen) diluted in 200µl of DMEM without supplements. To investigate the effect of different levels of *CYP26B1* variants, COS-1 cells were transfected with different concentrations and combinations of the full-length *CYP26B1*, or spliced variant construct or empty vector, with a final plasmid DNA concentration 1 µg.

For THP-1 cells, the cells were seeded in 12 wells plates at density of 1×10^6^ cells/well followed by differentiation with 20 nM PMA for 24 h. The next day, cells were transfected with 1 µg of either *CYP26B1* construct or pcDNA3.2/V5-DEST vector without insert using 2 µl of Turbofect (Fermentas, Leon-Rot, Germany).

In a second set of experiments, COS-1 cells were plated at 8×10^4^cells/well in 24-well plates, transfected with 50ng each of pTA-RARE-SEAP (Roche Diagnostics Scandinavia AB, Stockholm, Sweden) and pSG5-RARα constructs; 15ng of pMetLuc-Control (Invitro Sweden AB, Stockholm, Sweden) and 25 ng of pcDNA3.2/V5-DEST-CYP26B1 (spliced or full length ) or empty pcDNA3.2/V5-DEST, using 1.5µL of Lipofectamine 2000. After transfection, cells were incubated for 24 hours at 37°C with 5% CO_2_. Transfected cells were exposed to 1µM atRA or DMSO for 24 hours. The media were collected for analysis of secreted alkaline phosphatase (SEAP) activity after 24 hours incubation. The SEAP activity was measured using the SEAP Reporter gene Assay Chemiluminescence according to the manufacturer’s instruction (Roche AB). Secreted luciferase activity was measured using Ready-to-Glow™ Secreted Luciferase Reporter Assay kit Clontech (Invitro Sweden AB).

### Analysis of Retinoic Acid Metabolism in COS-1and THP-1 Cells by HPLC

COS-1 and THP1 cells were transfected for 24 hours with pcDNA3.2/V5-DEST-*CYP26B1*full length/spliced constructs and pcDNA3.2/V5-DEST empty vector as described above. The cells in each well were supplemented with 1 ml fresh DMEM containing 1µCi [^3^H]atRA (PerkinElmer Life Sciences, Boston, MA, USA). Following incubation with atRA for 1h the medium was removed, cells were washed once with ice-cold PBS containing 1mg/ml of bovine serum albumin, twice with ice-cold PBS without albumin and frozen at -20°C. Cell-associated radioactivity was measured as previously described [8]. In brief, radioactive cell content was separated by reversed-phase HPLC after hydrolysing the samples in 500 µl ethanol and 100 µl of 80% KOH followed by extraction of [^3^H]atRA with n-hexane. The extract was evaporated and dissolved in methanol, and subsequently separated on a 5-µm ODS column using a mobile phase containing acetonitrile/water/acetic acid (80∶20:0.05) at a flow rate of 0.8 mL/min. The eluate was mixed 1∶3 (vol/vol) with Optima Flow AP scintillation liquid and the radioactivity was measured online using a Radiometric 525 TR monitor (Canberra Packard). All experiments were conducted in a light-restricted regimen.

### Affinity Purification of CYP26B1

CYP26B1 was affinity purified from COS-1 cells transfected with the full length or spliced variant and from HUVEC and AOSMCs cells treated with 1µM of atRA for 48 hours, using mouse anti-human monoclonal CYP26B1 antibodies (Abnova, Taipei City, Taiwan) and the IP pierce kit (Fischer Scientific, Göteborg, Sweden) following the instruction from the company. Purified CYP26B1 full length and spliced variant were stored in a buffer (50mM KH_2_PO_4_, 50mM K_2_HPO_4_, 0.5 mM EDTA and 20% glycerol, pH 7.4) at -80°C.

### CYP26B1 Enzymatic Activity Assay

CYP26B1 assay was performed as follows: 5 nM of either full length or spliced CYP26B1, 10 nM oxidoreductase (Electra-Box Diagnostica AB, Tyresö, Sweden), 25nM [^3^H]atRA and 1 mM of NADPH were mixed. The total volume of the reaction was adjusted to 100µl with 100 mM pH 7.4 potassium phosphate buffer (KP_i_ buffer). The mixtures were incubated at 37°C for different times. The reactions were stopped by addition of 500 µl 99.5% ethanol followed by HPLC analysis as described above. The control used in the experiment was either the full length or spliced CYP26B1 without the addition of NADPH.

### Western Blot Analysis

Western blot was performed as previously described [28] using mouse anti- human CYP26B1 monoclonal antibodies (Abnova, Taipei City, Taiwan) at 1∶1000 dilution and horseradish-peroxidase-conjugated secondary antibodies anti-mouse 1∶1000 dilution, both dissolved in 3% non-fat dry milk (GE Healthcare, Uppsala, Sweden) in TBS. Peroxidase activity was detected using the ECL detection kit (GE Healthcare) and recorded on HyperFilm^TM^MP (GE Healthcare).

### Statistical Analysis

All data are presented as mean ± S.D., and comparisons between groups were analysed by paired Student’s t-test and one way ANOVA for comparing more than two groups. *P<0.05* was considered as statistically significant.
